# Trophectoderm biopsy of blastocysts for a preimplantation genetic test does not affect serum β-hCG levels in early pregnancy: a study using propensity score matching

**DOI:** 10.1186/s13048-021-00824-x

**Published:** 2021-06-11

**Authors:** Yixuan Wu, Ying Ying, Mingzhu Cao, Jianqiao Liu, Haiying Liu

**Affiliations:** 1 Department of Obstetrics and Gynecology, Center for Reproductive Medicine/Department of Fetal Medicine and Prenatal Diagnosis/BioResource Research Center, Key Laboratory for Major Obstetric Diseases of Guangdong Province, The Third Affiliated Hospital of Guangzhou Medical University, Guangzhou, China; 2 Key Laboratory of Reproductive Medicine of Guangdong Province, No. 63, Duobao Road, Guangzhou, Guangdong China; 3 Key Laboratory for Major Obstetric Diseases of Guangdong Province, No. 63, Duobao Road, Guangzhou, Guangdong China; 4 Key Laboratory of Reproduction and Genetics of Guangdong Higher Education Institutes, No. 63, Duobao Road, Guangzhou, Guangdong China

**Keywords:** Blastocyst, Human chorionic gonadotropin, Pregnancy, Preimplantation genetic test, Trophectoderm biopsy

## Abstract

**Background:**

Although preimplantation genetic test (PGT) has been used worldwide, few studies investigated the effect of trophectoderm biopsy of blastocysts on early embryo development. This study aimed to investigate whether trophectoderm (TE) biopsy of blastocysts for a PGT affected serum β-human chorionic gonadotropin (hCG) levels 14 days after transfer.

**Methods:**

This was a retrospective cohort study conducted at the Third Affiliated Hospital of Guangzhou Medical University. The study population comprised pregnant women undergoing the transfer of single vitrified-warmed blastocysts after PGT between January 1, 2018, and July 30, 2020. The control group had non-PGT cycles with other inclusion criteria identical to those for the study group. Propensity score matching was used to screen a group of patients so that the baseline characteristics were similar between the two groups. Serum β-hCG levels were compared between the PGT and non-PGT cycles. Multiple linear regression was used to analyze the influence of PGT on serum β-hCG levels, while receiver operating characteristic curves (ROC curves) were plotted to predict pregnancy outcomes using serum β-hCG levels.

**Results:**

Serum β-hCG levels were comparable between the PGT and non-PGT patients: live birth: 2503 ± 1702 mIU/mL vs 2266 ± 1289 mIU/mL (*P* = 0.219); clinical pregnancy: 2261 ± 1564 mIU/mL vs 2148 ± 1348 mIU/mL (*P* = 0.461); and ongoing pregnancy: 2412 ± 1589 mIU/mL vs 2278 ± 1308 mIU/mL (*P* = 0.422). Multiple linear regression analysis indicated no impact of PGT on the serum β-hCG level (standardized coefficient = − 0.001, *P* = 0.989). For clinical pregnancy, the cutoff value was 482 mIU/mL and 302 mIU/mL for PGT and non-PGT patients, respectively. The threshold to predict live birth was 1345 mIU/mL and 1621 mIU/mL in the PGT and non-PGT cycles, respectively.

**Conclusion:**

Trophectoderm biopsy of blastocysts for PGT did not affect the serum β-hCG level 14 days after transfer.

## Introduction

Preimplantation genetic test (PGT) has been used worldwide, with the main indications of monogenetic diseases, chromosomal abnormalities, recurrent pregnancy loss, and recurrent implantation failure. Removing 5–10 trophectoderm (TE) cells in the blastocyst stage is one of the procedures of PGT. However, few studies investigated the effect of TE cell reduction on early embryo development. Human chorionic gonadotropin (hCG), which represents the function of TE, is secreted by syncytiotrophoblast cells of the TE from the time of implantation.

A previous study on blastocysts cultured in vitro indicated that the mean cumulative hCG was inversely proportional to the number of removed TE cells. However, removing fewer than 10 TE cells did not significantly affect the secreted hCG levels [1]. Two other studies investigated the influence of PGT on the development of embryos in the early stage in vivo. Cho et al. explored the influence of blastomere biopsy on serum β-hCG levels of early pregnancy. They found that the mean serum β-hCG level was lower in PGT cycles than in intracytoplasmic sperm injection cycles for the fresh embryo transfer (ET). However, doubling times were comparable between the two groups on the post-ovulation day (POD) 12 and 21 [2]. Hobeika et al. found that the serum β-hCG level 9 days after blastocyst transfer was higher in PGT cycles than in non-PGT cycles [3]. Lu et al. found that the serum β-hCG levels 12 days after ET was significantly lower in the biopsy group than in the control group [4].

Early measurement of serum hCG level in patients undergoing PGT can predict the pregnancy outcomes earlier, thus reducing the anxiety of patients. In addition, it helps detect the very early biochemical pregnancy loss and evaluate the effect of biopsy on pregnancy outcomes more accurately.

However, in the study by Cho, the biopsy of blastomeres was performed in the cleavage stage, which was quite different from the current procedures in PGT. In addition, fresh embryos were not transferred on the same day after oocyte retrieval, which affected serum β-hCG levels [5]. In the study by Hobeika, one to two embryos were transferred, resulting in vanishing twin syndrome. Although the analysis was performed according to the number of embryos transferred, the sample size was relatively small, resulting in a bias. In the study by Lu et al., the baseline characteristics were different between the biopsy group and control group, affecting the serum β-hCG levels. Therefore, the present study aimed to investigate whether the TE biopsy of blastocysts for PGT affected serum β-hCG levels 14 days after transfer. Propensity score matching was used to screen a group of patients so that the baseline characteristics were similar between the two groups.

## Materials and methods

### Population

**Fig. 1:**
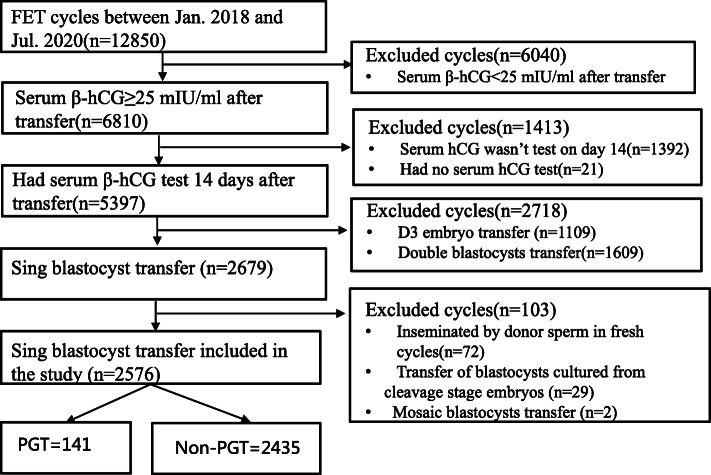
Process of data screening for analysis

This retrospective cohort study included PGT cycles with ET in the Department of Reproductive Medicine of the Third Affiliated Hospital, Guangzhou Medical University (Guangzhou, China) between January 1, 2018, and July 31, 2020. The control group involved the non-PGT cycles with other inclusion criteria identical to those for the study group. This study was approved by the ethics committee of the Third Affiliated Hospital of Guangzhou Medical University. The inclusion criteria were as follows: (1) serum β-hCG levels ≥25 mIU/mL 14 days after transfer; (2) single vitrified-warmed blastocyst transfer; (3) the day of vitrified the same as the day of transferred for all the blastocysts (Fig. 1). The exclusion criteria were as follows: (1) cycles with donor sperms (no oocytes donation in the center) because the age of the sperms might affect the outcomes of pregnancy [6]; (2) mosaic ET; and (3) cycles that transferred blastocysts cultured from frozen embryos in the cleavage stage (Fig. 1).

### Ovarian stimulation and oocyte retrieval

Controlled ovarian stimulation was performed using either long protocol or antagonist protocol. On days 2–4 of the cycle, 150–300 IU follicle-stimulating hormone (Gonal-F, Merck Serono, Italy) was used for ovarian stimulation. Oocyte maturation was triggered with 250 μg recombinant human chorionic gonadotropin (hCG) (Ovidrel, Merck Serono, Italy), and oocyte retrieval was performed 36 h later.

### Embryo culture and blastocyst biopsy

All the embryos were cultured to the blastocyte stage (5–6 days after oocyte retrieval), when the TE biopsy was performed on good- and fair-quality embryos. Good-quality embryos were defined as blastocysts of 3–6 AA/AB/BA/BB using the Gardner scoring system. Fair-quality embryos included blastocysts of 4–6 BC/CB [7]. The perforation of zona pellucida was induced with three to five laser pulses (Satum Active Laser System, RI, England). Five to 10 TE cells were aspirated with the biopsy pipet. After washing, the TE biopsy samples were placed in the polymerase chain reaction (PCR) tube with 2 μL of phosphate-buffered saline, centrifuged immediately, and stored in a refrigerator at − 80 °C for further processing. The biopsied blastocysts were vitrified using a kit (Kitazato Biopharma Co., Ltd., Shizuoka, Japan).

### Whole gene amplification and PGT

Whole gene amplification (WGA) of the biopsied samples and parental reference DNA was performed with amplification equipment (GeneQ, Hangzhou Bioer Technology, China) using a kit (SurePLEX WGA, Basecare, China). Next-generation sequencing and single nucleotide polymorphism (SNP) microarray were used for genome testing, with SNP microarray mainly applied for monogenetic diseases. The copy number variant (CNV) of 1.7–2.3 was defined as a normal region; CNV of 1.3–1.7 indicated a mosaic deletion region; CNV of 2.3–2.7 indicated a mosaic repeat region; CNV of greater than 2.7 indicated a repeat region; and CNV less than 1.3 indicated a deletion region. The minimum detectable CNV size was 4 MB, and the minimum mosaic CNV size was 10 MB. Data were processed using the Genome Studio program (iScan, Illumina, America).

### Frozen-thawed blastocyst transfer

Three protocols were used for endometrial preparation: natural cycle, artificial cycle, and ovarian stimulation cycle. Vaginal progesterone (Crinone, Merck Serono, England), 90 mg once a day, was applied for luteal-phase support. The serum β-hCG test was performed 14 days after ET, and luteal-phase support was continued to the tenth week in the case of intrauterine pregnancy.

### Hormonal assays

The immunochemiluminometric assay was performed for testing β-hCG (Architech i2000SR; Abbott Laboratories Inc., IL, USA). The range of detection was between 1.2 and 225,000 mIU/mL. The sensitivity of the assay was 1.2 mIU/mL, and the intraassay coefficient of variation was 7%.

### Definitions of pregnancy outcomes

Clinical pregnancy was defined as an intrauterine/extrauterine gestational sac detected by ultrasound with positive serum β-hCG. Biochemical pregnancy loss was defined as serum β-HCG level > 25 mIU/mL 14 days after transferring the embryo, which declined to < 5 mIU/mL at the end without any visible gestational sac on ultrasound. Early miscarriage was defined as fetal growth arrest or no cardiac activity detected in the gestational sac during the first 12 weeks of pregnancy. Ongoing pregnancy was defined as the pregnancy continued beyond 12 weeks with a live fetus. Live birth indicated pregnancy continued after 28 weeks of gestation with a live fetus.

### Statistical analysis

Since this was a retrospective study, the baseline characteristics were different between the PGT and non-PGT groups. Therefore, propensity score matching (PSM) was used to screen a group of patients so that the baseline characteristics were similar between the two groups. The multiple logistic regression model was used to calculate the propensity score, with PGT as the dependent variable and the variables in Table 1 as independent variables (female age, male age, duration of infertility, number of previous gestations and transfers, AMH level, BMI, endometrial thickness 5 days before transfer, and days of ET). The PSM was performed with 1:2 matching by the nearest neighbor matching, with the caliper width equal to 0.2 of the standard deviation (SD) of the logit of the propensity score (PS). SD was calculated for baseline variables before and after PSM; an absolute value lesser than 0.1 indicated a negligible imbalance.

**Table 1:** Characteristics of patients before and after PS matching

Characteristics	Before matching			After matching		
	PGT	Non-PGT	Standardized	PGT	Non-PGT	Standardized
	*N* = 141	*N* = 2435	difference	*N* = 138	*N* = 267	difference
Male age	32.5 ± 4.4	33.2 ± 4.9	− 0.154	32.4 ± 4.4	32.2 ± 4.5	0.034
Female age	30.5 ± 4.5	30.9 ± 4.1	− 0.084	30.4 ± 4.5	30.5 ± 4.1	− 0.01
No. of previous transfer	1.43 ± 1.28	1.76 ± 1.02	− 0.257	1.44 ± 1.30	1.51 ± 0.82	− 0.056
infertility duration (year)	2.9 ± 2.8	4.4 ± 2.8	− 0.534	3.0 ± 2.8	3.0 ± 2.3	−0.02
No. of previous gestation	1.55 ± 1.28	0.83 ± 1.06	0.411	1.44 ± 1.57	1.62 ± 1.40	−0.103
AMH (ng/mL)	5.35 ± 3.97	6.48 ± 4.47	− 0.284	5.41 ± 3.99	5.34 ± 3.78	0.018
BMI (kg/m^2^)	22.39 ± 3.09	22.15 ± 4.16	0.079	22.35 ± 3.10	22.65 ± 8.28	−0.097
EMT^a^ (mm)	8.7 ± 1.5	8.9 ± 1.6	− 0.114	8.7 ± 1.5	8.7 ± 1.5	0.024
Days of embryo transferred	5.30 ± 0.46	5.16 ± 0.37	0.303	5.28 ± 0.45	5.32 ± 0.47	−0.071
Day of embryo vitrified	5.30 ± 0.46	5.16 ± 0.37	0.303	5.28 ± 0.45	5.32 ± 0.47	−0.071
Day of embryo transferred %(*n*)			1.7E-5			
5	70.2 (99)	84.1 (2048)		71.7 (99)	70.4 (188)	0.781 ^b^
6	29.8 (42))	15.9 (387)		28.3 (39)	29.6 (79)	
Embryo morphology %(*n*)			0.940^b^			
Good	89.4 (126)	89.2 (2171)		89.1 (123)	90.6 (242)	0.630^b^
Fair	10.6 (15)	10.8 (264)		10.9 (15)	9.4 (25)	
PGT indications % (*n*)			NA^c^			NA^c^
Monogenetic diseases	45.4 (64)			45.7 (63)		
Chromosomal diseases	31.9 (45)			32.6 (45)		
Recurrent mscarriage	19.9 (28)			18.8 (26)		
Recurrent implantation failure	2.8 (4)			2.9 (4)		

^a^*EMT* endometrium thickness 5 days before transfer. ^b^Chi-square test. ^c^*NA* not applicable

Statistical analysis was performed using SPSS 22.0 software (IBM, NY, USA). Quantitative variables with homogenous variance were expressed as ^−^X ± SD, and the means were compared using the Students *t* test. A chi-squared test was used to compare rates. Multiple linear regression was applied to analyze the influence of PGT on serum β-hCG levels, while receiver operating characteristic curves (ROC curves) were plotted to predict pregnancy outcomes using serum β-hCG levels. A *P* value < 0.05 indicated a statistically significant difference.

## Results

### Baseline characteristics

A total of 141 PGT patients were included in the study, with 2435 non-PGT patients used as control. After the 1:2 PS matching, only 138 PGT patients and 267 non-PGT patients were included in the study. Most of the baseline characteristics were significantly different between the two groups before matching (Table 1). However, most of the aforementioned covariates were balanced between the two groups after matching, except the number of previous gestations. The indications of PGT were mainly monogenetic diseases (45.4%) and chromosomal diseases (31.9%). Recurrent miscarriage and implantation failure accounted for 19.9 and 2.8% of PGT, respectively (Table 1).

### Pregnancy outcomes

The rates of ongoing pregnancy in the PGT group were not significantly different from those in the non-PGT group (79.7% vs 79.4%, *P* = 0.942). The rates of early miscarriage (12.3% vs 16.5%) and ectopic pregnancy (0% vs 1.1%) were also comparable between the two groups (*P* > 0.05). However, the rate of biochemical pregnancy loss was significantly higher in the PGT group than in the non-PGT group (8.0% vs 3.0%, *P* = 0.025) (Table 2). For patients transferred with good-quality embryos, the rate of biochemical pregnancy loss was also significantly higher in the PGT group than that in the non-PGT group (8.1% vs 2.9%, *P* = 0.025). Though not statistically significant, the rates of biochemical pregnancy loss were higher for both the day 5 and 6 blastocyst transfer in the PGT group compared with those in the non-PGT group (6.1% vs 2.7 and 12.8% vs 3.8%) (Table 3).

**Table 2:** Pregnancy outcomes of patients after PGT v. non-PGT following PS matching

	PGT	non-PGT	***P***
Total ongoing pregnancy ^a^	79.7 (110)	79.4 (212)	0.942
Live birth^**b**^	57.2 (79)	68.9 (184)	
Ongoing pregnancy (following up)^c^	18.8 (26)	9.0 (24)	
Late miscarriage	3.6 (5)	1.5 (4)	
Early miscarriage	12.3 (17)	16.5 (44)	0.267
Biochemical pregnancy loss	8.0 (11)	3.0 (8)	0.025
Ectopic pregnancy	0 (0)	1.1 (3)	0.554^d^
Total	100.0 (138)	100.0 (267)	

Note: ^a^Total ongoing pregnancy = live birth + ongoing pregnancy (following up) + late miscarriage.

^b^Live birth only included patients transferred between January 2018 and March 2020.

^c^Ongoing pregnancy (following up) indicated gestations beyond 12 weeks, but the follow-up of live birth was not finished.

^d^Fisher Exact Test was used

**Table 3:** Relationships between embryos and rates of biochemical pregnancy loss

Embryo %(***n***)	PGT	non-PGT	***P***_**PGT vs non-PGT**_
**fair**	6.7 (1)	4.0 (1)	1.000^a^
**good**	8.1 (10)	2.9 (7)	0.025
***P***_**fair vs good**_	1.000^a^	0.550^a^	
**Days of embryo**			
**5**	6.1 (6)	2.7 (5)	0.197^a^
**6**	12.8 (5)	3.8 (3)	0.113^a^
***P***_**day 5 vs day 6**_	0.292^a^	0.698 ^a^	

Note: ^a^Fisher Exact Test was used. *P*_fair vs good_ indicated a comparison between fair embryos and good embryos

*P*_day 5 vs day 6_ indicated a comparison between day 5 embryos and day 6 embryos. *P*_PGT vs non-PGT_ indicated a comparison between PGT cycles and non-PGT cycles

### Primary outcomes

The serum β-hCG level of live birth in the PGT group was 2503 ± 1702 mIU/mL, which was not significantly different from that in the non-PGT group (2266 ± 1289 mIU/mL, *P* = 0.219). Serum β-hCG levels from cycles resulting in clinical pregnancy and ongoing pregnancy were comparable between the two groups: 2261 ± 1564 mIU/mL vs 2418 ± 1348 mIU/mL (*P* = 0.461) and 2412 ± 1589 mIU/mL vs 2278 ± 1308 mIU/mL (*P* = 0.422), respectively. The serum β-hCG level of early miscarriage in the PGT group was 1289 ± 951 mIU/mL, which was not significantly different from that in the non-PGT group (1652 ± 1377 mIU/mL, *P* = 0.324) (Table 4).

**Table 4:** Comparison of the serumβ-hCG level between patients of PGT vs non-PGT after PS matching

		PGT	Non-PGT	***P***
All pregnant patients	hCG	2096 ± 1603	2087 ± 1371	0.956
	*n*	138	267	
Live birth	hCG	2503 ± 1702	2266 ± 1289	0.219
	*n*	79	184	
Clinical pregnancy	hCG	2261 ± 1564	2148 ± 1348	0.461
	*n*	127	259	
Ongoing pregnancy	hCG	2412 ± 1589	2278 ± 1308	0.422
	*n*	110	212	
Early miscarriage	hCG	1289 ± 951	1652 ± 1377	0.324
	*n*	17	44	
Biochemical pregnancy loss	hCG	182 ± 130	129 ± 83	0.329
	*n*	11	8	

Note: Data of live birth included only those from January 2018 to March 2020

### Effect of PGT on serum β-hCG levels

Multiple linear regression analysis indicated that PGT was not related to the serum β-hCG level (Standardized coefficient = − 0.001, *P* = 0.989). Variables that affected the serum β-hCG level were BMI and days of ET (*P* = 0.026 and *P* = 0.01, respectively) (Table 5).

**Table 5:** Influence of PGT on the serum β-hCG level by multiple linear regression

	Unstandardized coefficient	Standardized coefficient	*t*	*P*
BMI	−22.720	−0.110	−2.236	0.026
Days of embryo transfer	− 421.551	− 0.132	− 2.555	0.01
PGT vs non-PGT^a^	− 2.016	−0.001	− 0.013	0.989

Note: Adjusted for female age, number of previous transfer, duration of infertility, AMH, BMI, endometrium thickness, days of embryo transfer, and morphology of the embryo transferred

### Prediction of pregnancy outcomes

**Fig. 2:**
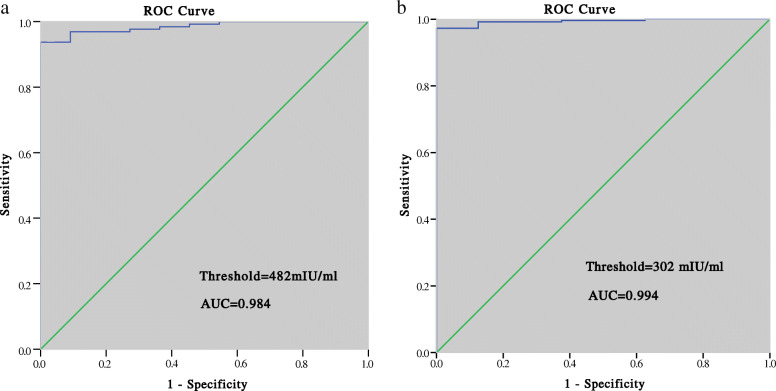
Prediction of clinical pregnancy by the serumβ-hCG level 14 days after blastocyst transfer: (**a**) PGT group; (**b**) non-PGT group

**Fig. 3:**
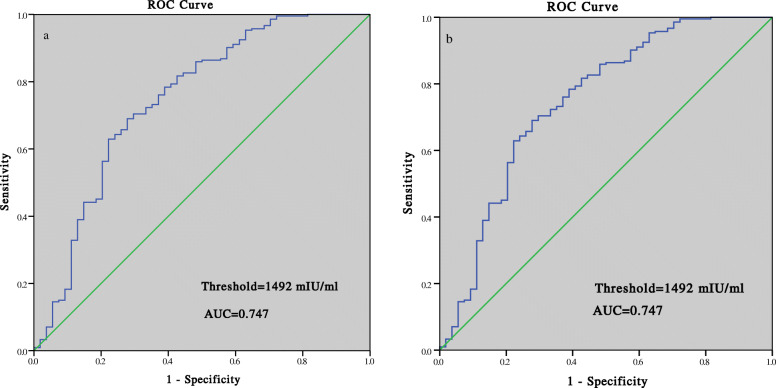
Prediction of ongoing pregnancy by the serumβ-hCG level 14 days after blastocyst transfer: (**a**) PGT group; (**b**) non-PGT group

**Fig. 4:**
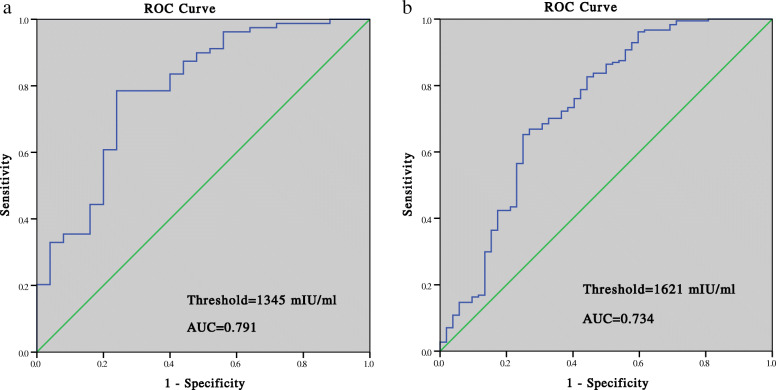
Prediction of live birth by the serumβ-hCG level 14 days after blastocyst transfer: (**a**) PGT group; (**b**) non-PGT group

The ROC curve analysis showed that for clinical pregnancy, the cutoff value was 482 mIU/mL for PGT patients, with the sensitivity of 93.7%, specificity of 100%, positive predictive value (PPV) of 100%, and area under the ROC curve (AUC) of 0.984. The threshold for clinical pregnancy in non-PGT cycles was 302 mIU/mL, with a sensitivity of 97.3%, specificity of 100%, PPV of 99.7%, and AUC of 0.994 (Fig. 2). For ongoing pregnancy, the threshold was 1328 mIU/mL in PGT cycles with the AUC of 0.842 and 1492 mIU/mL in non-PGT cycles with the AUC of 0.747 (Fig. 3). For live birth, the threshold was 1345 mIU/mL in PGT cycles with the sensitivity of 78.5%, specificity of 76.0%, and PPV of 90.1%, and 1621 mIU/mL in non-PGT cycles with the sensitivity of 65.2%, specificity of 75.0%, and PPV of 90.2% (Fig. 4).

### Live birth outcomes

The rate of cesarean section in the PGT group (50.0%) was not significantly lower than that in the non-PGT group (59.2%). The weight and height of newborns at birth were comparable between the two groups. Though not significantly different, the rates of premature birth and low birth weight (< 2.5 kg) were higher in the non-PGT group than in the PGT group. Although all the newborns from the non-PGT cycles were without birth defects, one birth defect was detected in the PGT group (Table 6).

**Table 6:** Live birth outcomes in PGT vs non-PGT patients

Parameters	PGT	non-PGT	***P***
*n*	78	184	
Types of delivery % (*n*)			0.168
Vaginal delivery	50.0 (39)	40.8 (75)	
Cesarean	50.0 (39)	59.2 (109)	
Birth weight (g)	3170 ± 511	3222 ± 502	0.453
Birth height (cm)	49.8 ± 2.5	49.6 ± 1.9	0.444
Gestational weeks	39.0 ± 1.2	39.1 ± 1.5	0.669
Sex % (*n*)			0.519
Male	50.0 (39)	45.7 (84)	
Female	50.0 (39)	54.3 (100)	
Premature birth % (*n*)	5.1 (4)	9.2 (17)	0.262
Birth weight < 2.5 kg % (*n*)	3.8 (3)	7.1 (13)	0.407^**a**^
Birth defects % (*n*)	1.3 (1)	0 (0)	0.298^**a**^

Note: ^a^Fisher exact test was used

## Discussion

The present study indicated that the trophectoderm biopsy of blastocysts for PGT did not affect the serum β-hCG level 14 days after transfer. This was confirmed by Dokras et al., who investigated the influence of TE biopsy on the development of blastocysts cultured in vitro. Their study showed that the removal of < 10 TE cells did not significantly decrease the amount of cumulative β-hCG secretion from day 3 to day 14 (87.6 ± 24.8 mlU/mL vs 146.2 ± 23.7 mlU/mL, *P* > 0.05) [1]. In the present study, only 5–10 TE cells were removed from the blastocysts for PGT. Therefore, the TE biopsy with < 10 cells had no significant influence on hCG secretion by embryos in the early stage.

A previous study by Cho et al. demonstrated that the removal of one to two blastomeres from embryos in the cleavage stage significantly decreased serum β-hCG levels in PGT patients on days 12, 14, and 21 after ovulation. However, the doubling time of serum β-hCG levels was comparable to that in the control group [2]. Lu et al. demonstrated that the trophectoderm biopsy decreased the serum hCG levels 12 days after blastocyst transfer (635.7 mIU/mL vs 720.2 mIU/mL, *P =* 0.005) [4]. However, in their study, baseline characteristics, such as the endometrium thickness on the transfer day, embryo quality, and endometrial preparation protocols, which might affect the serum hCG levels, were different between the biopsy and control groups. In contrast, Hobeika et al. found that the initial β-hCG level 9 days after the transfer was significantly higher in PGT cycles compared with that in the frozen ET (FET) and fresh cycles (182.4 mIU/mL vs 124.0 mIU/mL vs 87.1 mIU/mL, *P* < 0.05) [3].

The present study demonstrated that the mean serum β-hCG level 14 days after transfer in PGT cycles was not significantly different from that in non-PGT cycles. For each type of pregnancy outcome (biochemical pregnancy loss, early miscarriage, clinical pregnancy, and live birth), the β-hCG levels between the PGT and non-PGT cycles were similar. The multiple linear regression analysis further confirmed that PGT did not affect serum β-hCG levels. The factors that affected the serum β-hCG level were BMI and days of ET (day 5 vs day 6). Serum hCG levels reflect the developmental potential of embryos. This study found that BMI negatively correlated with serum hCG levels, suggesting that overweight or obesity had a negative impact on embryo quality. Cardozo et al. found that the increase in donor BMI was related to the decrease in the rates of clinical pregnancy and live birth, confirming that BMI had a negative impact on the quality of oocytes and embryos [8]. The study by Leary et al. demonstrated that the oocytes from overweight or obese women were smaller than those from normal-weight women. Meanwhile, the blastocysts from the overweight or obese women manifested reduced glucose consumption and increased endogenous triglyceride level. They postulated that the negative impact of obesity on oocytes might be associated with the decreased mitochondrial function [9]. The decline in oocyte quality leads to decreased embryonic development potential in the late stage, and hence a decrease in the serum hCG level.

The day 6 blastocysts had a lower β-hCG level for both PGT and non-PGT groups compared with day 5 blastocysts (PGT group: 1688 ± 1381 mIU/mL vs 2256 ± 1662 mIU/mL, *P* = 0.061; non-PGT group: 1774 ± 1357 mIU/mL vs 2218 ± 1359 mIU/mL, *P* = 0.016). This finding further indicated that the day 6 blastocysts were less potent than the day 5 blastocysts and thus grew slowly [10]. Previous studies did not adjust for the covariates that might have an impact on serum β-hCG levels. Therefore, the conclusion that PGT influenced serum β-hCG levels was not reliable. The present study proved that PGT did not influence serum β-hCG levels 14 days after single blastocyst transfer, following PSM and further adjustment for BMI and days of ET.

The rate of biochemical pregnancy loss was significantly higher in the PGT patients (8.0% vs 3.0%, *P* = 0.025). However, the days of ET and the quality of the embryo were similar in the two groups. After stratification by day of transfer and embryo quality, the tendency of the higher rate of biochemical pregnancy loss in the PGT group still existed (Table 3). The rate of biochemical pregnancy loss was 25% in the recurrent implantation failure (RIF) group and 11.5% in the recurrent miscarriage (RM) group, which was higher than that in the non-PGT group. The rate of biochemical pregnancy loss was 6.3 and 6.7% in patients with monogenetic disease and chromosome disease, respectively, which was not significantly higher than that in the non-PGT group. Therefore, the higher rate of biochemical pregnancy loss in the PGT group was mainly caused by the patients with RIF and RM, who had some underlying diseases that negatively affected live birth.

Previous studies indicated a significantly higher ongoing pregnancy rate and a lower miscarriage rate in PGT patients [11–13]. The present study showed that the rates of ongoing pregnancy and early miscarriage were not significantly different in PGT and non-PGT patients once pregnancy was achieved (serum β-hCG ≥25 mIU/mL). The explanation for this finding was that non-PGT patients in the present study were young (mean age: 30.5 years) with the transfer of mainly D5 blastocysts (70.4%), resulting in a higher pregnancy rate and a lower miscarriage rate.

The cutoff value for clinical pregnancy 14 days after the transfer was 482 mIU/mL in PGT cycles, which was higher than that in non-PGT cycles (302 mIU/mL). This might be explained by the higher rate of biochemical pregnancy loss in the PGT group (8.0% vs 3.0, *P* = 0.026), which might have resulted from the perforation of zona pellucida during blastocyst biopsy. The thresholds predicting ongoing pregnancy and live birth were lower in the PGT group than in the non-PGT group: 1328 mIU/mL vs 1492 mIU/mL and 1345 mIU/mL vs 1621 mIU/mL, respectively. Lu et al. also demonstrated that PGT patients had a lower threshold of serum β-hCG levels for live birth than the control group (368.55 mIU/mL vs 411.45 mIU/mL) 12 days after transfer [4].

The present study had the following advantages. First, PSM was used to screen a group of patients so that the baseline characteristics were similar between the two groups, thus significantly decreasing the bias that affected serum β-hCG levels. Second, only a single blastocyst transfer was included in the study, avoiding the vanishing twin syndrome [14]. Third, all the patients had the serum β-hCG test on exactly the same day after ET in a single laboratory, minimizing the bias from the blood test. However, the study had the following disadvantages. The sample size in the PGT group was relatively small, which might have limited the analysis of the effect of PGT on early embryo development. Second, the serum β-hCG test was performed on the 14th day after transfer, which might not be applicable to analyze the effect of PGT on embryo development earlier than this stage [15]. Third, to evaluate the effect of biopsy on the blastocysts, the best way is to compare the serum β-hCG between euploid blastocysts with TE biopsy and euploid blastocysts without biopsy, or non-PGT blastocysts with TE biopsy and non-PGT blastocysts without TE biopsy. However, this cannot be performed in the clinic.

## Conclusion

Trophectoderm biopsy of blastocysts for PGT did not affect serum β-hCG levels in early pregnancy.

## Data Availability

The data sets used and/or analyzed in the present study are available from the corresponding author on reasonable request.

## Abbreviations

AMH: Anti-Müllerian hormone
AUC: Area under the ROC curve
CI: Confidence interval
CNV: Copy number of variants
ET: Embryo transfer
HCG: Human chorionic gonadotropin
NPV: Negative predictive value
PGT: Preimplantation genetic test
PCR: Polymerase chain reaction
PPV: Positive predictive value
PSM: Propensity score matching
ROC: Receiver operating characteristic
SD: Standard deviation
SNP: Single nucleotide polymorphism
TE: Trophectoderm
WGA: Whole gene amplification
